# Bilingualism and cognition: The role of language use and proficiency in healthy older adults

**DOI:** 10.1002/alz70857_103020

**Published:** 2025-12-25

**Authors:** Sergio Grueso, Gonzalo Sánchez‐Benavides, Oriol Grau‐Rivera, Miguel A Santos‐Santos, Laia Subirats, Ferran Prados Carrasco, Marco Calabria

**Affiliations:** ^1^ NeuroADaS Lab Universitat Oberta de Catalunya, Barcelona, Spain; ^2^ Barcelonaβeta Brain Research Center (BBRC), Barcelona, Spain; ^3^ Hospital del Mar Research Institute (IMIM), Barcelona, Spain; ^4^ Sant Pau Memory Unit, Department of Neurology, Hospital de la Santa Creu i Sant Pau, Biomedical Research Institute Sant Pau ‐ Universitat Autònoma de Barcelona, Barcelona, Spain; ^5^ UCL Hawkes Institute University College London, London, United Kingdom; ^6^ Queen Square Multiple Sclerosis Centre, Department of Neuroinflammation, UCL Queen Square Institute of Neurology, London, United Kingdom

## Abstract

**Background:**

Many studies suggest bilingualism as a cognitive reserve factor, but the evidence of such bilingual advantage is still controversial. Additionally, there is limited knowledge about which type of bilingual experience has the greatest impact on cognitive decline associated with aging, making it difficult to explain the effect of bilingualism on delaying the symptoms of dementia.

**Method:**

To understand the impact of language experience on cognitive aging, we compared the cognitive performance of 2502 cognitively unimpaired individuals aged 44–74 years old from the Alzheimer's and Families (ALFA) study, considering their age of language acquisition (AoA) and active use of both languages (Spanish as L1 and Catalan as L2). We defined three groups based on L2 AoA and proficiency: early high‐proficient bilinguals (*n* = 1515), late high‐proficient bilinguals (*n* = 708) and late low‐proficient bilinguals, primarily Spanish‐dominant (*n* = 279).

**Result:**

On tests assessing non‐verbal reasoning and processing speed (measured by WAIS‐IV Matrix Reasoning and Coding, respectively), high‐proficiency bilinguals, whether early (*p* < 0.001 for both tests) or late (*p* < 0.001 for Coding and *p* =  0.002 for Matrix Reasoning), outperformed late low‐proficient bilinguals, even after controlling for age, education, gender, and other cognitive reserve factors such as occupational complexity, social and physical activities, and cultural and intellectual pursuits. The performance of the two high‐proficient groups was comparable across all cognitive test scores. Early high‐proficient bilinguals also outperformed late low‐proficient bilinguals in visuospatial processing (WAIS‐IV Visual Puzzles; *p* =  0.028) and working memory, attention and encoding (WAIS‐IV Digits Span, *p* < 0.001). In the case of late high‐proficient bilinguals, they outperformed late low‐proficient bilinguals in Semantic verbal fluency (*p* = 0.013). The three groups did not show significant differences in general cognitive decline as measured by MMSE (*p* = 0.384) or episodic verbal memory measured with Memory Binding Test (*p* > 0.05 in all conditions).

**Conclusion:**

This suggests that high L2 proficiency and L1‐L2 active use is associated with better cognitive performance, irrespective of AoA. Furthermore, the cognitive benefits of actively speaking a second language seems to be limited to non‐linguistic functions, with no significant effect on verbal episodic memory or lexical retrieval.

